# A retrospective cohort study to investigate the incidence of cancer-related weight loss during chemotherapy in gastric cancer patients

**DOI:** 10.1007/s00520-020-05479-w

**Published:** 2020-05-03

**Authors:** Masaru Fukahori, Masayuki Shibata, Satoshi Hamauchi, Eiji Kasamatsu, Koji Machii

**Affiliations:** 1 Multidisciplinary Treatment Cancer Center, Kurume University Hospital, 67, Asahi-machi, Kurume-shi, Fukuoka, 830-0011 Japan; 2 Division of Gastrointestinal Oncology, Shizuoka Cancer Center, Shizuoka, Japan; 3 Medical Affairs Department, Ono Pharmaceutical Co., Ltd., Osaka, Japan

**Keywords:** Cancer-related weight loss, Incidence, Survival, Gastric cancer, Adverse events

## Abstract

**Purpose:**

This study aimed to evaluate cancer-related weight loss (WL) after the start of first-line chemotherapy as a surrogate marker for cancer cachexia in patients with advanced gastric cancer. We investigated the incidence of WL and the relationship between WL and overall survival (OS) or adverse events.

**Methods:**

We conducted a retrospective cohort study in 131 patients with advanced gastric cancer who received first-line systemic chemotherapy between September 1, 2010, and August 31, 2016, at Kurume University Hospital and Shizuoka Cancer Center Hospital. WL was defined in this study as weight loss of > 5% or weight loss of > 2% with a body mass index of < 20 kg/m^2^ within the last 6 months after the start of chemotherapy.

**Results:**

Median age and median Eastern Cooperative Oncology Group performance status of the patients participating in this study were 68 years old and 0, respectively. Incidence of WL was 53% at the first 12 weeks after starting first-line chemotherapy, and increased to 88% after 48 weeks. Overall survival rates were significantly associated with WL at 12, 24, and 48 weeks. Appetite loss and fatigue were more frequent and more severe in patients with WL.

**Conclusion:**

WL was especially observed in more than half the patients within 12 weeks after starting chemotherapy. WL appeared to relate to adverse events or reduced survival. These results suggest the importance of monitoring WL or providing nutritional support at the beginning of chemotherapy.

**Electronic supplementary material:**

The online version of this article (10.1007/s00520-020-05479-w) contains supplementary material, which is available to authorized users.

## Introduction

Cachexia is a multifactorial malnutrition syndrome that is typically observed in patients with chronic disease, and especially in patients with cancer [1]. The European Palliative Care Research Collaborative (EPCRC) proposed three criteria to define cancer cachexia [2]. Three criteria are based on patients’ weight loss over a 6-month period, and two of these consider either low body mass index (BMI) or diagnosis of sarcopenia. Although cancer-related weight loss (WL) is a primary characteristic, conventional nutritional support fails to reverse WL in many cases. Cancer cachexia eventually leads to impairment in the activities of daily living due to loss of skeletal muscle [1, 3–10]. Therefore, understanding of this syndrome is essential to address the unmet clinical needs. The number of patients with gastric cancer in Japan is decreasing, but gastric cancer is still the fifth most frequent cancer. The prevalence of gastric cancer is also relatively high in East Asia compared to other regions. The primary treatment in gastric cancer is surgical resection. Patients with advanced or metastatic gastric cancer are treated with systemic chemotherapy with platinum agents and fluoropyrimidines being the most common first-line treatments. It points to cancer cachexia as frequent disease in patients with advanced gastric cancer [11–15]. Cancer cachexia and its associated metabolic changes may decrease tolerance for cancer therapies, in particular cytotoxic chemotherapy. For instance, in patients with non-small cell lung cancer (NSCLC), the prevalence of cachexia defined by the EPCRC criteria was more than 20% within 12 weeks after starting chemotherapy. The study also showed that NSCLC patients with cachexia have relatively lower quality of life (QOL) and shorter survival [16]. In gastrointestinal cancer, some studies based on non-EPCRC criteria investigated the correlation of WL with prognosis and symptoms, and showed that overall survival (OS) was significantly associated with WL at the time of the diagnosis of advanced gastric cancer. However, there is limited information regarding WL after starting chemotherapy. In this context, we performed a retrospective study to estimate the incidence of WL that developed after the start of first-line chemotherapy as a surrogate marker for cancer cachexia in patients with advanced gastric cancer. Moreover, we evaluated the relationships between WL and incidence of AEs, overall survival (OS), treatment status, or laboratory values to clarify the influence of WL on gastric cancer patients receiving cancer chemotherapy.

## Methods

### Ethics

This study was approved by the ethics review committees of Kurume University Hospital (reference 18,076) and Shizuoka Cancer Center (reference T30-31-30-1-7). This study was registered on the University Hospital Medical Information Network Clinical Trials Registry (UMIN000033693).

### Patients

We searched medical record databases at the study centers to identify patients who were diagnosed with advanced gastric cancer and underwent first-line systemic chemotherapy between September 1, 2010, and August 31, 2016, at Kurume University Hospital and Shizuoka Cancer Center Hospital.

### Definition of WL

We employed two of the EPCRC criteria to define WL in this study, either weight loss of > 5% or weight loss of > 2% with a BMI of < 20 kg/m^2^ within the last 6 months because there were no written records for sarcopenia. The start date of chemotherapy was recorded as the index date.

### Data collection

Patients’ body weights, laboratory test data, and categories and grades of AEs based on Common Terminology Criteria for Adverse Events version 5.0 were collected from the start of chemotherapy through 156 weeks. The lowest weights every 4 weeks, the latest laboratory tests, and the highest-grade AE data at each observation period (0, 1–12, 13–24, 25–36, and 37–48 weeks, and beyond 48 weeks) were collected.

### Data analyses

Primary endpoints were the time when each patient first developed WL and the cumulative incidence of WL over the whole observation period. Regarding cumulative incidence, death events were not considered as censored in this analysis. Secondary endpoints were the relationship between WL and incidence of AEs, OS, treatment status, and laboratory tests comparing patients with and without WL. OS was calculated from the beginning of first-line chemotherapy, and assessed using the Kaplan–Meier method. The differences in OS were evaluated using the log-rank test between patients without WL and those who developed WL within 12, 24, and 48 weeks after starting first-line chemotherapy. All *P* values were two-sided, and *P* < 0.05 was considered as statistically significant. Hazard ratios and 95% confidence intervals (CI) of presence or absence of WL for OS were evaluated using the Cox proportional hazards model with or without adjusted model by stratified by number of metastases, Eastern Cooperative Oncology Group (ECOG) performance status (PS), and alkaline phosphatase (ALP). These factors were set based on the multivariate analysis of data from the JCOG9912 trial for patients with advanced gastric cancer in Japan [17]. The cut-off time for occurrence of WL was 12 weeks because the number of patients with WL was similar in patients without WL. Median survival times with 95% CI were determined using the Brookmeyer and Crowley method. Cumulative incidence of AEs within 24 and 48 weeks after starting first-line chemotherapy was evaluated. Appetite loss and fatigue were chosen as AEs. Data analyses were performed using SAS for Windows version 9.4 or later (SAS Institute, Cary, NC, USA).

## Results

### Patients

A total of 1032 patients with advanced gastric cancer were identified at our two institutions during the 6-year period. However, only 131 patients met the all inclusion criteria and were included in the study, and 901 were excluded, predominantly because body weight data were not available. Of the patients included in the study, 100 were men (76.3%), and 31 were women (23.7%); median age, 68 years old (range, 28–84); median BMI, 21.2 kg/m^2^ (13.6–37.3); and median ECOG PS, 0 (0–2) (Table 1). The primary sites of cancer were the gastroesophageal junction (12.2%), body (69.5%), and pylorus (18.3%), and most patients had stage IV disease (99.2%). First-line chemotherapy regimens were S-1 + cisplatin/S-1 + oxaliplatin/capecitabine + oxaliplatin/S-1 + cisplatin + trastuzumab (61.8%), capecitabine + oxaliplatin + trastuzumab/S-1 + oxaliplatin + trastuzumab (12.2%), or S-1/capecitabine (15.3%). The median neutrophil/lymphocyte ratio, C-reactive protein (CRP), albumin, and hemoglobin were 3.22 (0.86–48.01), 0.58 mg/L (0.01–13.76), 3.74 g/dL (1.69–4.90), and 12.0 g/dL (6.9–16.3), respectively. Patient characteristics are summarized in Table 1. There are no remarkable differences in characteristics between patients with or without WL at 12 weeks after starting chemotherapy except that the median values of CA19–9 were slightly different (35.60 vs 12.65, respectively).

**Table 1:** Patient characteristics

Characteristics	Patients (*N* = 131)	Patients with WL at 12w (*N* = 70)	Patients without WL at 12w (*N* = 61)
Sex			
Male	100 (76.3)	50 (71.4)	50 (82.0)
Female	31 (23.7)	20 (28.6)	11 (18.0)
Age (years)	68.0 (28–84)	68.0 (33–84)	67.0 (28–84)
BMI (kg/m^2^)	21.16 (13.6–37.3)	20.64 (15.35–37.32)	21.68 (13.62–27.77)
ECOG PS	0 (0–2)	0 (0–2)	0 (0–2)
0	71 (54.2)	35 (50.0)	36 (59.0)
1	49 (37.4)	28 (40.0)	21 (34.4)
2	11 (8.4)	7 (10.0)	4 (6.6)
Primary site			
Gastroesophageal junction	16 (12.2)	7 (10.0)	9 (14.8)
Body	91 (69.5)	51 (72.9)	40 (65.6)
Pylorus	24 (18.3)	12 (17.1)	12 (19.7)
UICC stage			
III	1 (0.8)	0 (0)	1 (0.8)
IV	130 (99.2)	70 (100)	60 (98.4)
First-line chemotherapy			
SP/SOX/CapeOX/SPT	81 (61.8)	48 (68.6)	33 (54.1)
CapeOX + Tmab/SOX + Tmab	16 (12.2)	6 (8.6)	10 (16.4)
S-1/capecitabine	20 (15.3)	11 (15.7)	9 (14.8)
Others	14 (10.7)	5 (7.1)	9 (14.8)
Pathological classification			
Intestinal type (tub, pap)	57 (43.5)	29 (41.4)	28 (45.9)
Diffuse type (por, sig)	73 (55.7)	41 (58.6)	32 (52.5)
Other	1 (0.8)	0 (0)	1 (1.6)
Neutrophil/lymphocyte ratio	3.209 (0.86–48.01)	3.182 (1.25–48.01)	3.256 (0.86–13.65)
CA19–9, ng/mL	21.30 (0.6–18,575.0)	35.60 (2.0–18,575.0)	12.65 (0.6–7322.0)
Albumin, g/dL	3.740 (1.69–4.90)	3.640 (1.69–4.70)	3.800 (2.30–4.90)
CRP, mg/L	0.580 (0.01–13.76)	0.845 (0.01–9.89)	0.570 (0.03–13.76)
Hemoglobin, g/dL	12.00 (6.9–16.3)	12.10 (6.9–15.2)	11.80 (7.6–16.3)

Values are *n* (%) or median (range)

*BMI* body mass index, *ECOG PS* Eastern Cooperative Oncology Group performance status, *UICC* Union for International Cancer Control, *SP* S-1 + cisplatin, *SOX* S-1 + oxaliplatin, *CapeOX* capecitabine + oxaliplatin, *SPT* S-1 + cisplatin + trastuzumab, *por* poorly differentiated adenocarcinoma, *sig* signet ring cell carcinoma, *tub* tubular adenocarcinoma, *pap* papillary adenocarcinoma, *CA19–9* carbohydrate antigen 19–9, *CRP* C-reactive protein

### Onset of WL

**Fig. 1:**
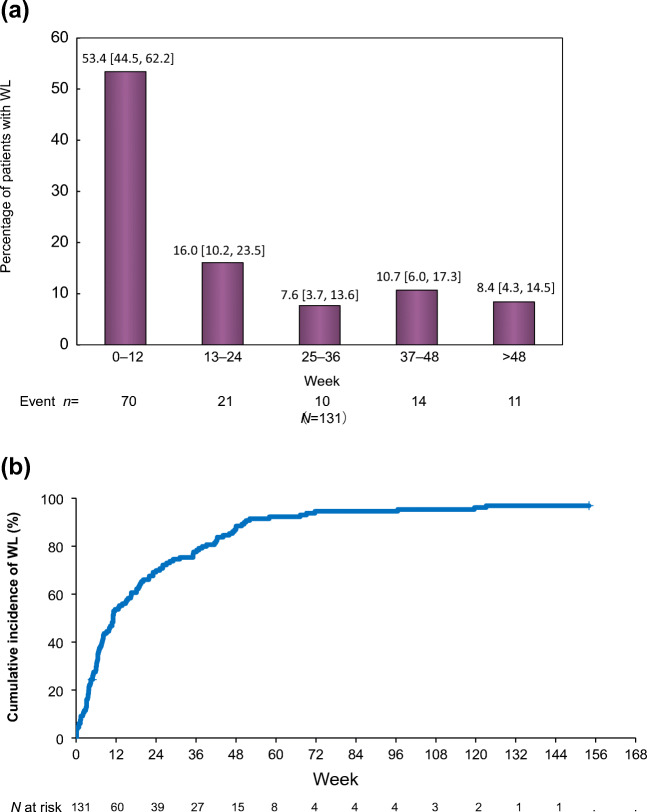
**a** Time of onset of WL after starting first-line chemotherapy. Percent of incidence of WL in each bar are noted and 95% confidence intervals are noted in brackets. **b** Cumulative incidence of WL after starting first-line chemotherapy. *N* = 131

Time of WL onset (1–12, 13–24, 25–36, 37–48 weeks, and beyond 48 weeks) is shown in Fig. 1a. In all, 53.4% (95% CI 44.5–62.2) patients experienced WL within 12 weeks after starting chemotherapy. The cumulative incidence of WL after the start of first-line chemotherapy reached 87.7% at 48 weeks, and 96.1% over the whole study period (Fig. 1b).

### Survival

**Fig. 2:**
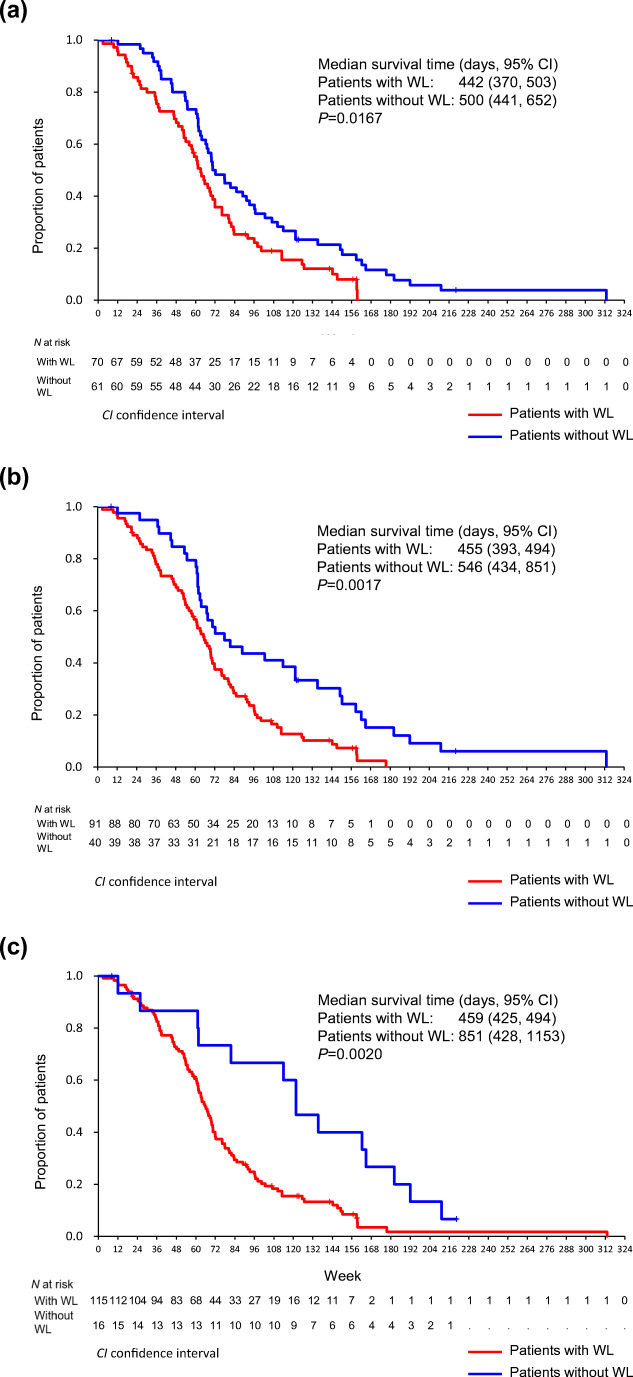
**a** Overall survival according to presence or absence of WL within 12 weeks after the start of chemotherapy. **b** Overall survival according to presence or absence of WL within 24 weeks after the start of chemotherapy. **c** Overall survival according to presence or absence of WL within 48 weeks after the start of chemotherapy *CI* confidence interval

The OS rates of patients who experienced WL or not within 12, 24, and 48 weeks of starting chemotherapy are shown in Fig. 2. Landmark analyses were also performed at these time points. The OS rates were significantly different between patients with and without WL in the 12-week analysis (log-rank *P* = 0.0167) (Fig. 2a). The median survival time was 442 (370–503) days for patients with WL versus 500 (441–652) days for patients without. The 12-week landmark analysis also showed significantly shorter OS in patients with WL (log-rank *P* = 0.0312). Significant differences in the OS rates were also observed when patients were grouped according to the presence of WL within 24 weeks (Fig. 2b) and 48 weeks (Fig. 2c) after starting chemotherapy. The hazard ratio for OS of patients with and without WL within 12 weeks after starting first-line chemotherapy was 1.51 (1.04–2.21) based on the unadjusted model, whereas it was 1.39 (0.95–2.05) for OS based on the adjusted model. The hazard ratio (95% CI) for OS of patients with ECOG PS 1 and 2 over those with ECOG PS 0 was 1.53 (1.04–2.26) based on the adjusted model (Table 2). Median survival time after the onset of WL was 368 (311–420) days.

**Table 2:** Prognostic factors for overall survival

	HR (95% CI)
Presence of WL within 12 weeks of starting treatment	1.39 (0.95–2.05)
ECOG PS (> 1 vs 0)	1.53 (1.04–2.26)
Number of metastases	1.25 (0.95–1.64)
ALP (> 359 U/L vs < 359 U/L)	1.14 (0.73–1.76)

*HR* hazard ratio, *CI* confidence interval, *ECOG PS* Eastern Cooperative Oncology Group performance status, *ALP* alkaline phosphatase

### AEs in patients with or without WL

**Fig. 3:**
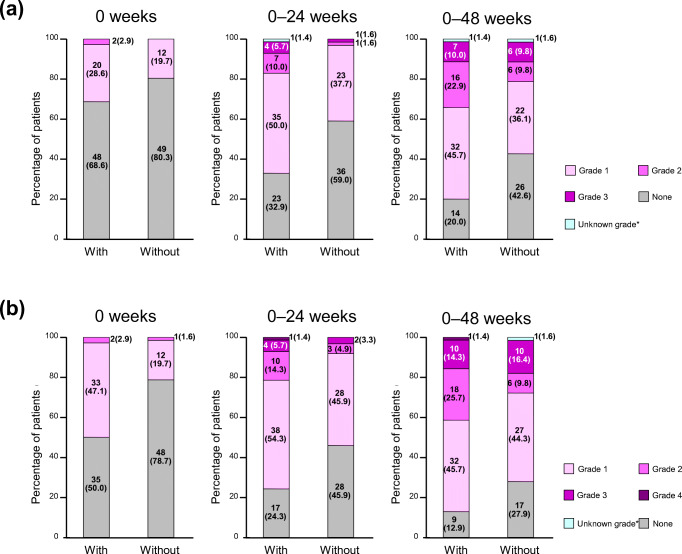
**a** Fatigue and **b** appetite loss in patients with or without WL within 12 weeks after the start of chemotherapy. **a** *1 patient with WL and 1 without WL after the start of chemotherapy experienced an unknown grade of fatigue. **b** *1 patient without WL after the start of chemotherapy experienced an unknown grade of appetite loss

The presence and severity of appetite loss and fatigue in patients with and without WL occurring within 12 weeks after the start of first-line chemotherapy are shown in Fig. 3. The proportions of patients with WL and ≥ grade 2 severe fatigue were 2.9% at baseline (week 0) and 34.3% from baseline to 48 weeks (Fig. 3a), compared with 0% and 21.2%, respectively, at the same time points in patients without WL. Severe appetite loss (≥ grade 2) was shown in a higher proportion of patients with WL from baseline to 48 weeks than in patients without (Fig. 3b).

### Laboratory values

Laboratory test values were apparently independent of WL that developed within 12 weeks after starting first-line chemotherapy. Among patients with WL, the mean value of albumin slightly decreased by about 0.33 mg/dL from baseline to 48 weeks. A similar decrease of albumin was also observed in patients without WL. Likewise, there were no significant differences in other laboratory tests, including CRP, and neutrophil and lymphocyte counts in patients with and without WL (Table S1).

## Discussion

We conducted this retrospective study to survey the development of WL, which was defined using a part of EPCRC criteria, during chemotherapy in patients with advanced gastric cancer. A key finding of this study was the high rate of WL in this cohort of patients. Indeed, approximately half of the patients experienced WL within 12 weeks after starting first-line chemotherapy, and this increased to 87.7% by 48 weeks.

The OS rates were significantly affected by WL that developed within 12, 24, and 48 weeks. Our landmark analyses also showed that the OS was significantly different between patients with and without WL. These results strongly suggest that onset of WL is as a prognostic factor for poor OS in patients with advanced gastric cancer. However, the adjusted hazard ratio for OS of patients developing WL within 12 weeks showed no statistical significance although the hazard ratio for the OS evaluated using the unadjusted model was statistically significant. This discrepancy could be attributed to the limited number of patients in this study; therefore, further analysis with an increased number of patients is required for accurate evaluation.

Appetite loss and fatigue were chosen as AEs of interest in this study, because Takayama et al. reported that these were the most relevant AEs in cancer cachexia in their investigation of the relationship between WL and QOL in patients with lung cancer using MD Anderson Symptom Inventory (Japan) [16]. In this study, the cumulative incidence of appetite loss and fatigue increased irrespective of WL. However, higher incidences and worse grades of appetite loss and fatigue were observed in patients with WL. Therefore, we emphasized the results of gastric cancer were similar to those of lung cancer for appetite loss and fatigue. These AEs apparently lead to interference of chemotherapy tolerance and have a negative impact on the patient’s wellbeing and QOL. Although the cause–effect relationship between cachexia and onsets of appetite loss and fatigue is not clear, monitoring WL during chemotherapy can be a tool to predict the development of these AEs.

As described above, WL likely results in poor prognosis or worse grades of appetite loss and fatigue. Thus, it is important to monitor body weight in patients with gastric cancer carefully after first-line treatment. Moreover, preventing WL through providing nutritional support during chemotherapy may lead to prolonging OS and to decreasing severe AEs [18]. Further research is needed to prevent WL.

In this study, we could not evaluate WL before starting chemotherapy due to limited available information regarding patients’ pre-visit weights. However, in pancreatic ductal adenocarcinoma (PDAC), the incidence of WL > 5% over up to 6 months prior to diagnosis was 63%, and the WL was found to be a prognostic factor for reduced overall survival (OS) [19]. In gastric cancer, severe postoperative WL, which is closely related with poor S-1 compliance, is an important risk factor for survival [20]. As the measurement of bodyweight is simple, easy, and practical [21], further studies on WL from pre-visit to chemotherapy might increase the awareness of healthcare professionals and patients that measurement of weight is important and necessary.

### Limitations

This study had some limitations, including its retrospective design. The small number of patients may have limited proper assessment of the correlation between WL and OS in the secondary endpoints. In addition, although we found higher rates of AEs during chemotherapy, the current study design did not allow us to determine whether WL leads to AEs, or vice versa.

It should be noted that we used only two of the EPCRC criteria but not the third (sarcopenia and > 2% weight loss). It has been shown that 12.6% of patients with lung cancer and cachexia were matched only to the third criterion [22], suggesting that the number of patients with WL in this study is underestimated as patients with cachexia.

We evaluated WL after starting chemotherapy but not before. This is partly because the importance of body weight changes before visiting a clinic has not been recognized among healthcare professionals or patients in Japan so that limited data for pre-visit body weight were available. Some patients may already experience WL at the start of chemotherapy, which could not be evaluated in this study.

Other AEs were not evaluated because the patients received a wide variety of regimens, each associated with a range of AEs, and this made meaningful analysis difficult.

## Conclusions

WL during chemotherapy was especially observed within 12 weeks after starting chemotherapy in patients with advanced gastric cancer. WL within 12 weeks appeared to relate to AEs or reduced survival. These results suggest the importance of monitoring WL or providing nutritional support at the beginning of chemotherapy.

## Electronic supplementary material

ESM 1 (PDF 272 kb)

ESM 2 (DOCX 19 kb)
