# Supplementary figures and images for: A retrospective cohort study to investigate the incidence of cancer-related weight loss during chemotherapy in gastric cancer patients
Source: Support Care Cancer. 2020 May 3;29(1):341–8. doi: 10.1007/s00520-020-05479-w (PMC7686005; doi:10.1007/s00520-020-05479-w)

# Supplementary Fig. 1

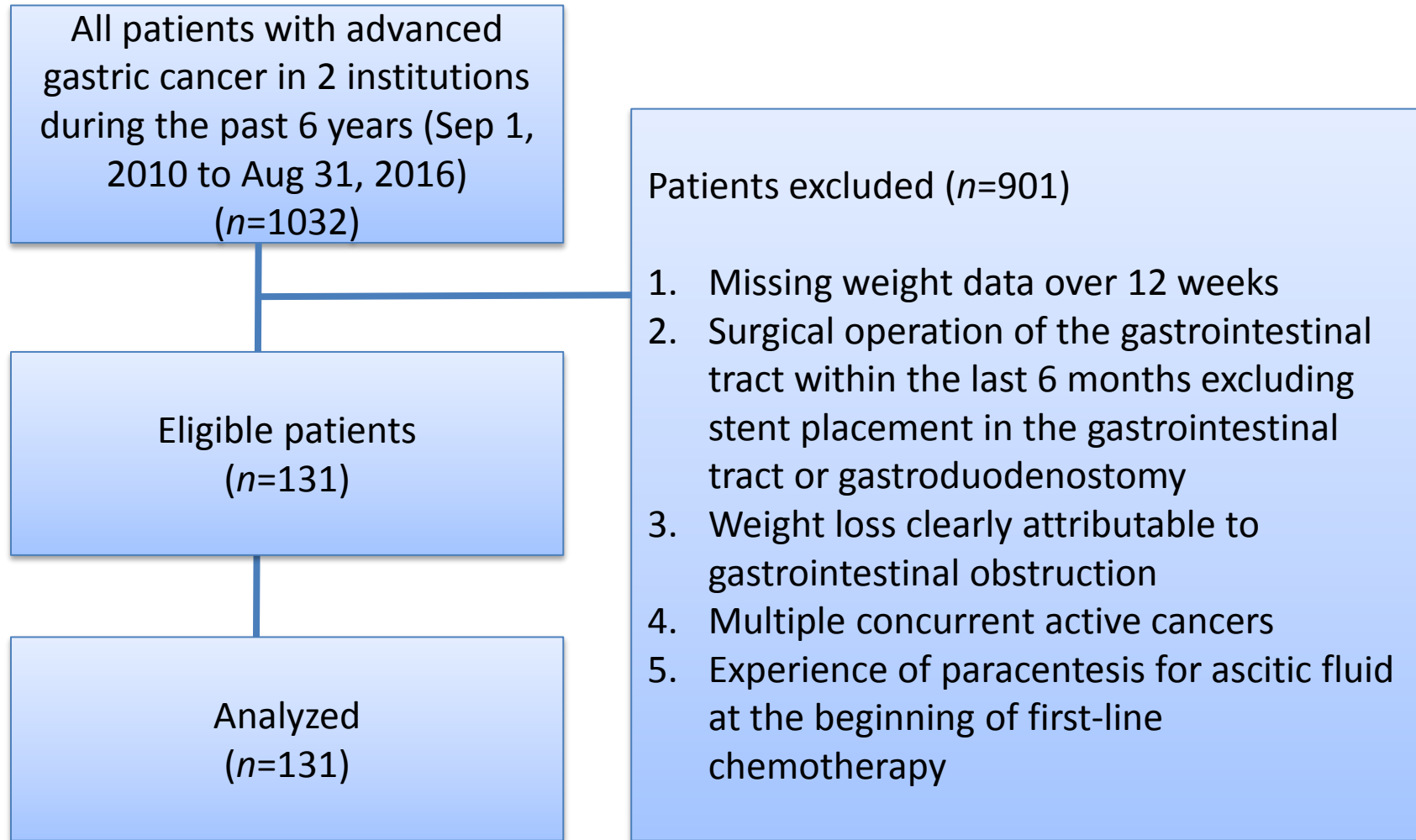

Supplement: Supplementary file 1 — (PDF 272 kb) [file 520_2020_5479_MOESM1_ESM.pdf]
