# Supplementary material for: A retrospective cohort study to investigate the incidence of cancer-related weight loss during chemotherapy in gastric cancer patients
Source: Support Care Cancer. 2020 May 3;29(1):341–8. doi: 10.1007/s00520-020-05479-w (PMC7686005; doi:10.1007/s00520-020-05479-w)
Supplement: Supplementary file 2 — (DOCX 19 kb) [file 520_2020_5479_MOESM2_ESM.docx]

# Supplementary materials

**Table S1.**Correlation between the presence or absence of WL within 12 weeks after the start of first-line chemotherapy and the change from baseline in selected laboratory values

|  | With WL (*n*=70) | | | | | | | | | | Without WL (*n*=61) | | | | | |  |  |
| --- | --- | --- | --- | --- | --- | --- | --- | --- | --- | --- | --- | --- | --- | --- | --- | --- | --- | --- |
|  | *n* | | median | | mean | | min | | max | SD | *n* | median | mean | min | max | SD | *P* value |  |
| CRP (mg/dL) | | | | | | | | | | | | | | | | | |  |
| 12W | | 66 | | −0.175 | | −0.663 | | −5.75 | 10.07 | 2.541 | 59 | −0.320 | −0.734 | −12.11 | 5.82 | 2.240 | 0.8698 | |
| 24W | | 60 | | −0.095 | | −0.441 | | −7.07 | 14.55 | 2.888 | 59 | −0.090 | −0.418 | −13.48 | 5.44 | 2.476 | 0.9639 | |
| 48W | | 50 | | 0.020 | | 0.255 | | −6.81 | 10.41 | 2.900 | 49 | 0.000 | 0.119 | −7.52 | 4.22 | 1.989 | 0.7856 | |
| Albumin (g/dL) | | | | | | | | | | | | | | | | | | |
| 12W | | 66 | | −0.10 | | −0.13 | | −1.8 | 1.5 | 0.62 | 59 | 0.07 | 0.11 | −0.6 | 1.6 | 0.44 | 0.0178 | |
| 24W | | 60 | | −0.15 | | −0.19 | | −2.0 | 1.1 | 0.64 | 59 | −0.09 | −0.04 | −1.1 | 1.8 | 0.57 | 0.1592 | |
| 48W | | 50 | | −0.30 | | −0.33 | | −1.8 | 1.3 | 0.68 | 49 | −0.30 | −0.28 | −2.0 | 2.1 | 0.70 | 0.7007 | |
| Neutrophils (/μL) | | | | | | | | | | | | | | | | | | |
| 12W | | 65 | | −1815.0 | | −2857.3 | | −68874 | 5256 | 8562.2 | 57 | −1944.0 | −2524.3 | −11143 | 2545 | 2504.7 | 0.7776 | |
| 24W | | 58 | | −2007.5 | | −3450.5 | | −67946 | 557 | 8806.8 | 58 | −1950.0 | −2103.5 | −7978 | 3773 | 2397.2 | 0.2634 | |
| 48W | | 50 | | −1151.0 | | −2090.7 | | −67691 | 12515 | 9927.3 | 48 | −1946.5 | −1777.9 | −12446 | 5285 | 2636.1 | 0.8332 | |
| Total lymphocytes (/μL) | | | | | | | | | | | | | | | | | | |
| 12W | | 66 | | 103.0 | | 59.4 | | −1129 | 1475 | 541.0 | 58 | −73.0 | 11.0 | −961 | 1480 | 456.2 | 0.5942 | |
| 24W | | 60 | | −86.5 | | -41.0 | | −1060 | 1770 | 623.3 | 58 | −88.5 | −48.3 | −729 | 1027 | 388.4 | 0.9390 | |
| 48W | | 50 | | −163.0 | | −163.1 | | −1701 | 4938 | 942.0 | 48 | −180.5 | −167.3 | −1412 | 1984 | 589.2 | 0.9791 | |

*SD* standard deviation; *CRP* C-reactive protein; *W* weeks
